# COSMO-RS Solubility Screening and Coumarin Extraction from *Pterocaulon polystachyum* with Deep Eutectic Solvents

**DOI:** 10.3390/molecules30173468

**Published:** 2025-08-23

**Authors:** Victor Hugo Rodrigues, Arthur Cavassa, Júlia Cardeal, Nathalya Brazil, Helder Teixeira, Gilsane von Poser, Rubem Mário Vargas, Ana Rita Duarte, Eduardo Cassel

**Affiliations:** 1 Laboratório de Operações Unitárias, Escola Politécnica, Pontifícia Universidade Católica do Rio Grande do Sul, Porto Alegre 90619-900, RS, Brazil; victor.rodrigues94@edu.pucrs.br (V.H.R.);; 2 Programa de Pós-Graduação em Ciências Farmacêuticas, Universidade Federal do Rio Grande do Sul, Porto Alegre 90010-150, RS, Brazil; ntbrazil@gmail.com (N.B.); helder.teixeira@ufrgs.br (H.T.);; 3 LAQV-REQUIMTE, Faculdade de Ciências e Tecnologia, Universidade Nova de Lisboa, 2829-516 Caparica, Portugal; ard08968@fct.unl.pt

**Keywords:** predictive model, relative solubility, coumarin, emerging solvents

## Abstract

Deep eutectic solvents (DESs) have been studied to obtain extracts from medicinal plants, aiming for a more environmentally friendly process. Aligned with this initiative, the use of predictive thermodynamic models for screening the best solvent represents a theoretical action to reduce experimental time and cost. Therefore, this study aimed to perform and validate a relative solubility screening of 5-methoxy-6,7-methylenedioxycoumarin and prenyletin-methyl-ether at 313 K in choline chloride, menthol, and betaine-based DES, using the COSMO-RS model in COSMOThermX software. The density of DES was also predicted with a maximum error of 7.31% for this property. Ultrasound-assisted extraction (UAE) with DES at 313 K, 30 min, and a solid/liquid ratio of 1:20 (*w*/*w*) was performed to confirm the theoretical solubility results experimentally, as the extracts were analyzed through ultrafast liquid chromatography (UFLC) for coumarin content. For the results, the coumarin molecules presented intense peaks in the nonpolar region of their σ-profile, and the relative solubility screening indicated the DES Men/Lau (2:1), known for its hydrophobic nature and low polarity, as the best DES to solubilize these coumarins. Nevertheless, the UFLC results, and the complementary solubility screening of pigments, showed an interaction preference of this DES with chlorophylls instead of coumarins. This result was corroborated by spectrophotometric analysis of the extracts in UV-Vis, demonstrating that experimental validation is still mandatory in extraction processes and that predictive methodologies such as COSMO-RS should be used as guiding tools and analyzed in a greater context, considering the complexity of plant matrices in the beginning of simulations.

## 1. Introduction

The search for eco-friendly and sustainable extraction processes is within the concept of green chemistry, which demands the reduction or removal of the synthesis, production, and application of chemical products that can be dangerous to human health and the environment, as well as reducing costs, risks, and environmental impacts [1]. Replacing traditional solvents used in extraction processes for emerging solvents helps to consolidate this search [2]. In this context, eutectic mixtures based on hydrogen-bonding interaction between one hydrogen-bond acceptor (HBA) and one or more hydrogen-bond donors (HBD), such as quaternary ammonium salts, amides, organic acids, and polyalcohols, have been studied as alternative solvents, named as deep eutectic solvents (DESs) [3].

DESs do not require complex preparation processes and present a low production and operation cost, especially when compared to other emerging solvents, such as ionic liquids, and can be designed as active solvents in the extraction process without the need for downstream purification steps [4]. Thermodynamically, DESs are liquid solvents at lower temperatures than for an ideal solution [5], which can ensure the non-degradation of thermolabile phenolic compounds in the extraction process [6]. Owing to these properties and advantages, several studies reported the use of DES as an alternative solvent to obtain natural compounds from plant matrices, preserving the bioactive properties of the target compounds [7].

Coumarins are natural compounds of interest to the food and pharmaceutical industries. They play a vital role in plant and animal biology with antioxidant, antimicrobial, anti-inflammatory, and antifungal activities. They have been considered the main bioactive substances of the genus *Pterocaulon* [8], including the *P. polystachyum* species, found in some South America countries like Brazil, Argentina, and Paraguay. The 5-methoxy-6,7-methylenedioxy and 7-(3-methyl-2-butenyloxy)-6-methoxy coumarins are two examples of these substances already identified in the extracts of *P. polystachyum* from previous studies [9]. The first coumarin demonstrated a cytotoxic effect on glioma and leukemia cells, while the second one, trivially named prenyletin-methyl-ether, in a mixture with another chemically related coumarin (prenyletin), presented antifungal activity against *Cryptococcus neoformans* and *Microsporum gypseum* [10]. Additionally, both compounds have been tested together in a mixture of coumarins for cytotoxicity against bladder tumor cell lineage [9].

Finding the best extraction solvent to obtain these compounds is a challenge, and the COSMO-RS model has been used as a predictive tool for selecting and screening the best DES to be used in solid–liquid extraction processes, aiming to reduce experimental efforts due to the numerous combinations possible between HBA and HBD to form different DESs [11]. Although computational software allows the scanning of various mixtures, experimental validation is still necessary to ensure good predictive accuracy [12]. Another issue related to accurate predictivity in solid–liquid extractions using DESs is the role of water in the mixture, usually presented due to the hygroscopic nature of DES constituents [13] or added to reduce viscosity [14]. They can form supramolecular complexes and therefore should be considered as such in the simulation, using a pseudomolecular approach [15], which is computationally more demanding but may improve model predictivity.

COSMO for real solvents (COSMO-RS) [16] was published as an extension of the original COSMO model [17], combining results from quantum chemistry with statistical thermodynamics to describe interactions between surface charges, enabling the estimation of different thermodynamic properties of pure and in-mixture compounds. Its main advantage lies in its independence from experimental data, qualitative precision in estimating properties such as vapor pressure and solubility, and the ability to differentiate isomers of molecules [18]. In the COSMO model, Coulomb charges are virtually placed on the surface part of its molecular structure and are subsequently optimized for a minimum energy [19], thus being considered a variant of the dielectric continuum solvation models of apparent surface charge [18]. A charge density surface and a σ-profile are generated, with the latter defined as the probability of a segment of the molecular surface having a specific charge, considering the contributions of electrostatic misfit, hydrogen bonds, and van der Waals energies, which are unique to each compound and allow predictive calculations [20].

Due to the advantages of using DESs in extraction processes, the possible applications for coumarin extracts, and to help consolidate the use of predictive tools applied to methods of obtaining natural products, this study aimed to predict the solubility of the 5-methoxy-6,7-methylenedioxycoumarin and prenyletin-methyl-ether at 313 K in eight different choline chloride, menthol, and betaine-based DESs, using the COSMO-RS model applied in the COSMOthermX software to scan for the best solvent to solubilize these coumarins. No predictive models and computational tools have been applied to obtain these specific compounds to date. Ultrasound-assisted deep eutectic solvent extraction from the aerial parts of *P. polystachyum* was performed and the obtained extracts were analyzed regarding the coumarin content obtained through ultrafast liquid chromatography. The results were compared with the predicted values from COSMO-RS to verify the reliability of the method applied, validate the procedure, and suggest future improvements.

## 2. Results and Discussion

### 2.1. Coumarin Molecule Optimization

**Figure 1:**
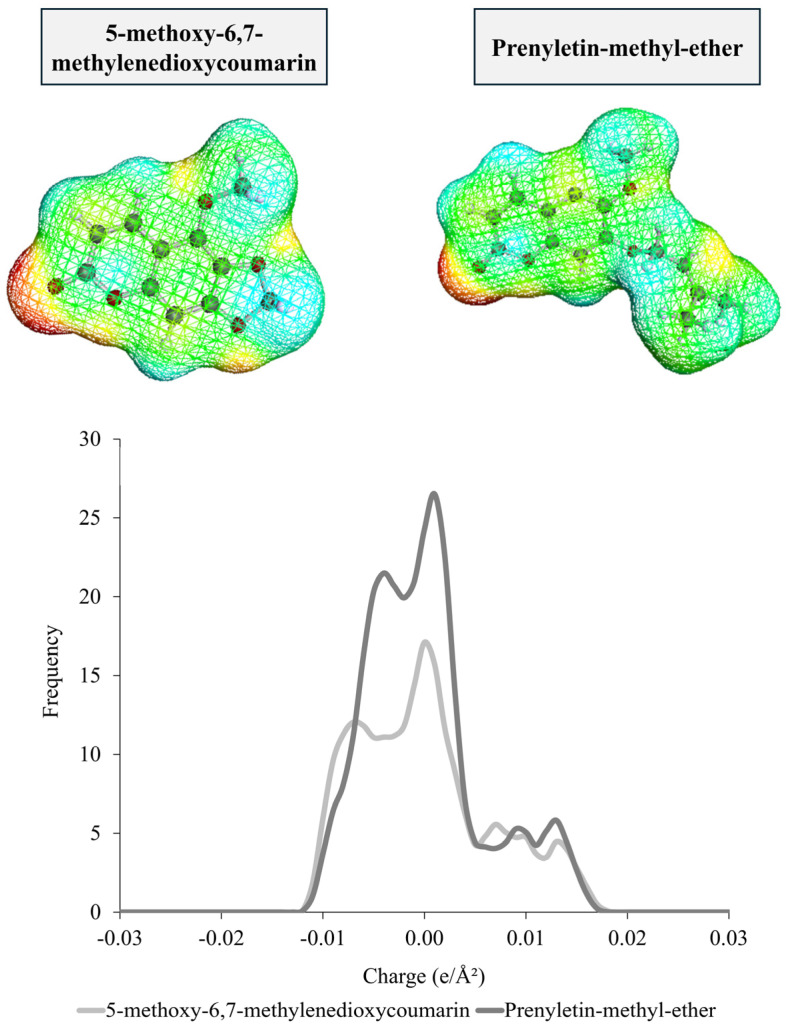
Charge density surface and the generated σ-profiles of 5-methoxy-6,7-methylenedioxycoumarin and prenyletin-methyl-ether.

The charge density surface and σ-profile of 5-methoxy-6,7-methylenedioxycoumarin and prenyletin-methyl-ether (Figure 1) can be analyzed as a first approach of the solubility screening since they give information about the non-polar and polar regions of the molecules and can be used to predict how the intermolecular interactions between two or more chemical species occur. For these coumarins, a polar region with a positive charge (red color) is observed in the charge density surface due to the presence of a lactone group, characteristic of coumarin molecules. Still, the structure is characterized by non-polar regions (green color) that represent neutrally charged zones due to aromatic rings, which is also observed in the σ-profiles by an intense peak in the non-polar region (0.00 e/Â^2^). This indicates that solvents with lower polarity are recommended for extracting these compounds.

### 2.2. Experimental and Predictive DES Density Comparisons

Before the solubility screening, the density (ρ) of all DESs at 313 K was predicted in COSMOthermX, and the results are presented in Table 1. The goal was to compare the results with already published values to verify if the approach chosen to represent the DESs (electroneutral mixture) was accurate. For the statistical analysis, the absolute deviation (*AD*) and error (%) were calculated considering the difference between experimental (*exp*) and predicted (*pred*) values.

The smallest deviations were observed for lactic acid/glycine/water (3:1:3) and menthol/lauric acid (2:1) DESs, with percentage errors of less than 1%, while betaine-based DESs presented the largest absolute deviations, which represented an error of 7.31% for betaine/glycerol (1:3) DES. It can be associated with the TurbomoleX optimization of this molecule, which could be further explored and refined. In addition, a gap in the interactions between betaine and compounds with alcohol function (DES 5, 6, and 8) was observed since the deviation for DES 7, which contains the acid HBD, was less pronounced. As COSMO-RS is a fully predictive model that only considers the molecular structure of each compound, an error of less than 8% was considered acceptable [26] for the approach chosen. Nonetheless, this error is specific to density and cannot be extrapolated to other properties, which must be validated on a case-by-case basis.

### 2.3. Coumarins Solubility Screening

**Figure 2:**
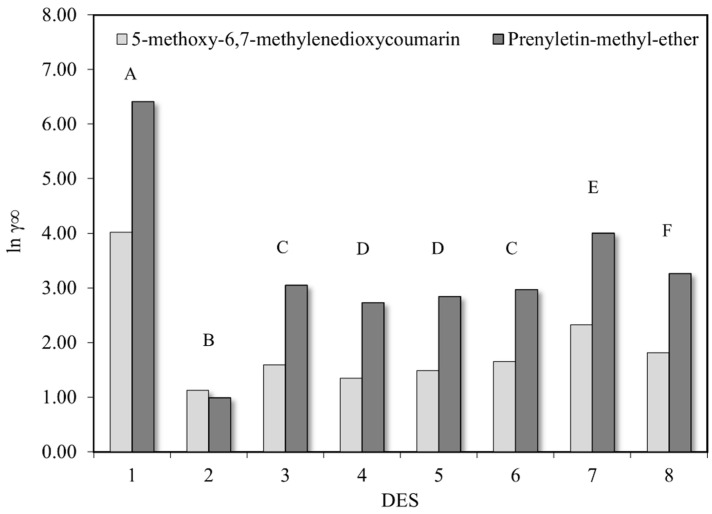
Absolute logarithmic activity coefficient at infinite dilution of 5-methoxy-6,7-methylenedioxycoumarin and prenyletin-methyl-ether in 8 different DESs at 40 °C. Equal capital letters (A–F) indicate a group result similarity above a 95% confidence level.

The results of solubility screening performed in COSMOthermX are presented in Figure 2, where eight different DESs were evaluated to find the best one to extract 5-methoxy-6,7-methylenedioxycoumarin and prenyletin-methyl-ether from *P. polystachyum.* Complete-linkage clustering was performed with Euclidean distance to assess the similarity between the DES predicted solubility values for both coumarins. In this analysis, group results with a similarity level above 95% are clustered and indicated with the same capital letter in Figure 2, where six distinct groups named from “A” to “F” are represented. Group C (DES 3 and DES 6) and group D (DES 4 and DES 5) presented a similarity level of 98.35% and 97.09%, respectively.

The logarithmic activity coefficient values for both coumarins in DES 2 remained at the same level and close to 1.00, while they increased for prenyletin-methyl-ether compared to 5-methoxy-6,7-methylenedioxycoumarin in all the other DESs, showing more affinity between DES 2 with the molecular structure of coumarins, regardless of which one. In the work of Zurob et al. [27], a direct relation between the solubility of compounds and the simulated activity coefficient at infinite dilution in DES performed in COSMOthermX was explored, showing that lower values for the activity coefficient meant greater probability of interactions between solvent and solutes, increasing the extraction capacity. Therefore, the results presented in Figure 2 indicated DES 2 (menthol/lauric acid 2:1) as one of the best to solubilize both coumarins. Furthermore, the σ-profile analysis denoted a strong interaction between these compounds and a lower polarity solvent, as is the case of DES 2, in addition to a hydrophobic nature, as experimentally determined in the work of Rebocho et al. (2022) [21].

### 2.4. Extraction of Coumarins from P. polystachyum

Experimental extraction of coumarins from *P. polystachyum* with the screened DESs was performed to validate the predictive results. However, it is reported that some DESs, namely DES 5, DES 6, and DES 7, present high viscosity [24,28], which hinders mass transfer during solid–liquid contact [29]. One way to overcome this extraction limitation is to add water [30,31] in their composition. Nevertheless, the coumarin molecules present in this plant are lipophilic, as shown in their charge density surfaces and σ-profiles, and this would not be beneficial for the extraction of these compounds. This is demonstrated by the solubility screening results for DES 1, a solvent with water as an intrinsic part of its composition, and that presented the higher activity coefficients at infinite dilution. The addition of water to a DES also increases its polarity [32], which is not desirable. Furthermore, based on prediction values for the logarithmic activity coefficient, DES 5 was clustered with DES 4, representing statistically one group with a similarity level above 95%, the same for DES 6 and DES 3. Considering the whole context presented, especially the viscosity, DES 1, DES 5, DES 6, and DES 7 were discarded for the experimental extraction assays. The remaining DES 2, 3, 4, and 8 were used for UAE at 313 K, 30 min, and an S/L ratio of 1:20. The extracts obtained were filtered and analyzed through ultrafast liquid chromatography for coumarin content.

UFLC was performed with the primary goal of verifying if it was possible to obtain the target coumarins from UAE with DESs and to compare experimental and predictive results in a relative scale. For identification of the coumarins, the retention times and UV absorption spectra were compared with previous reports of extracts from *P. polystachyum* [9].

**Figure 3:**
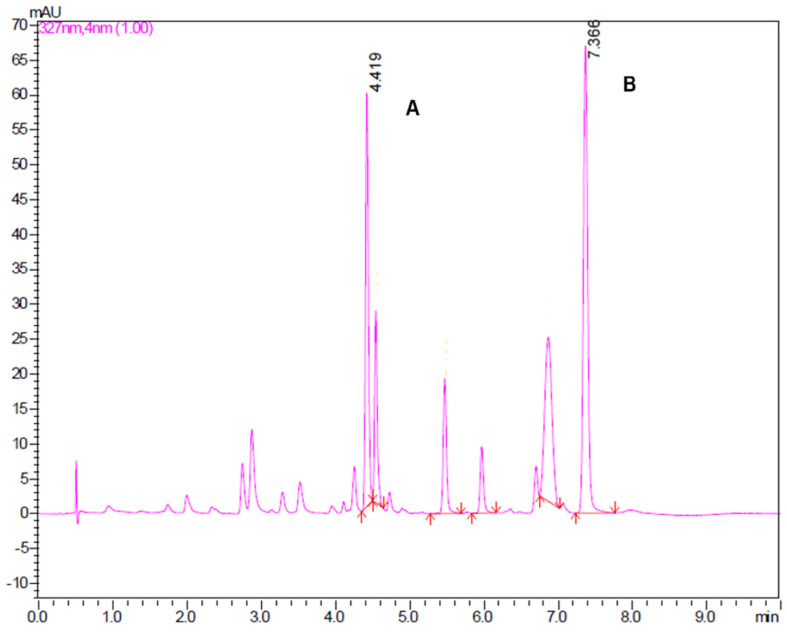
UFCL chromatogram profile of UAE DES 2 extract, highlighting peaks A (5-methoxy-6,7-methylenedioxycoumarin) and B (prenyletin-methyl-ether). Red arrows indicate the beginning and end of chromatographic peaks.

**Figure 4:**
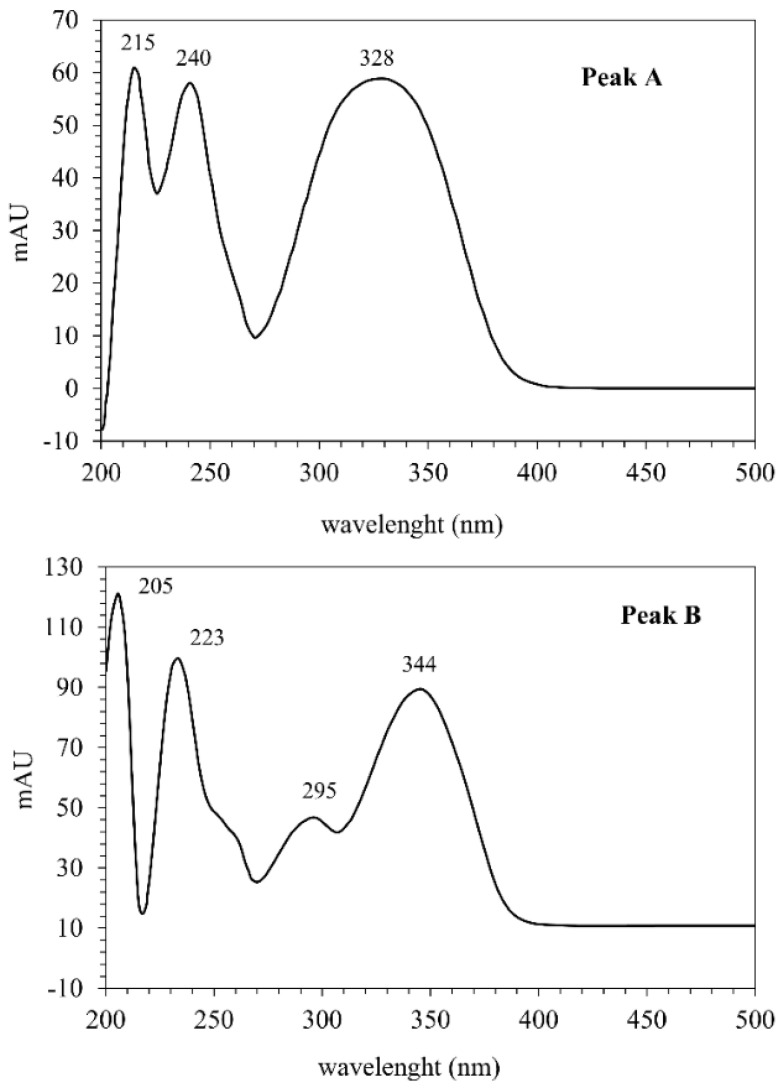
UV absorption spectra of peaks A (5-methoxy-6,7-methylenedioxycoumarin) and B (prenyletin-methyl-ether) of the DES 2 extract.

For the DES 2 extract, the chromatogram profile is presented in Figure 3, highlighting two peaks with a retention time of 4.419 min (peak A) and 7.366 min (peak B), attributed to 5-methoxy-6,7-methylenedioxycoumarin and prenyletin-methyl-ether, respectively. UV absorption spectra of peaks A and B (Figure 4) are very alike with those reported by Scopel et al. [9] from a qualitative point of view, allowing to infer the presence of the target compounds in the DES extracts. Both coumarins have been found as the main compounds in *P. polystachyum* extracts obtained by different extraction methods [10,33]. For the remaining DES extracts, the same pattern is observed in the chromatogram profile and UV absorption spectra, and they are presented in the Supplementary Material (Figures S1–S6). One of the main advantages of an extraction process using DESs is that they can be included with the target compounds in the final formulation desired to enhance stability and bioactivity of the extracts, avoiding the need for further purification steps [4]. Future studies should explore this possibility for therapeutic applications with the extracts obtained in this manuscript.

Chromatographic areas of peaks A and B for all DES extracts were calculated and are presented in Table 2. According to the relative scale results, DES 2 was the second and third best solvent in the solubilization of 5-methoxy-6,7-methylenedioxycoumarin and prenyletin-methyl-ether, respectively, while DES 8 showed the higher coumarin content. In this analysis, it is expected that higher chromatographic areas mean higher content of these coumarins in the DES. These experimental results are not in agreement with those predicted in the solubility screening, which indicated DES 2 as the best solvent to solubilize both coumarins. The main hypothesis for this disagreement is that the hydrophobic DES 2 had a preference solvent/solute interaction with other compounds during UAE, namely chlorophylls *a* and *b*, not predicted in COSMOThermX solubility screening, which was performed considering one solute at a time. This DES was reported in the work of Rebocho et al. [21] as selective for chlorophylls in the fractioned extraction of mate tea leaves. This statement highlights that the COSMO-RS model needs to be improved and evaluated in a condition where multiple solutes compete for interaction with the solvent, if one seeks to simulate the extraction process of natural compounds.

The increase in chlorophyll and pigment extraction is observed with decreasing polarity of the solvents [35], while polar DES can limit the chlorophyll solubility [36]. Additionally, the length of the HBD chain used to form the DES can directly affect the interaction with chlorophylls. Singh et al. [37] reported that longer alkyl chains in the HBD affect the hydrophobic character of the DES, resulting in a greater extraction of the chlorophylls. Since DES 2 has a hydrophobic nature, formed by the mixture of menthol as HBA and lauric acid as HBD, which contains longer alkyl chains than the other compounds used as HBD in this work (glycerol and ethylene glycol), DES 2 is expected to extract higher amounts of chlorophylls than other DESs and to have a preferential interaction with the pigments instead of other compounds.

Furthermore, during sample preparation for UFLC analysis, DES 2 extract formed two phases in dilution with the aqueous solution of acetonitrile, the mobile phase used in the method developed and standardized for the analysis of coumarins from *Pterocaulon* species, due to the hydrophobic nature of the DES. This behavior was also reported in the work of Ribeiro et al. [38], which may have impacted the coumarins chromatographic areas detected by the analysis.

### 2.5. Chlorophylls Solubility Screening in DESs

**Figure 5:**
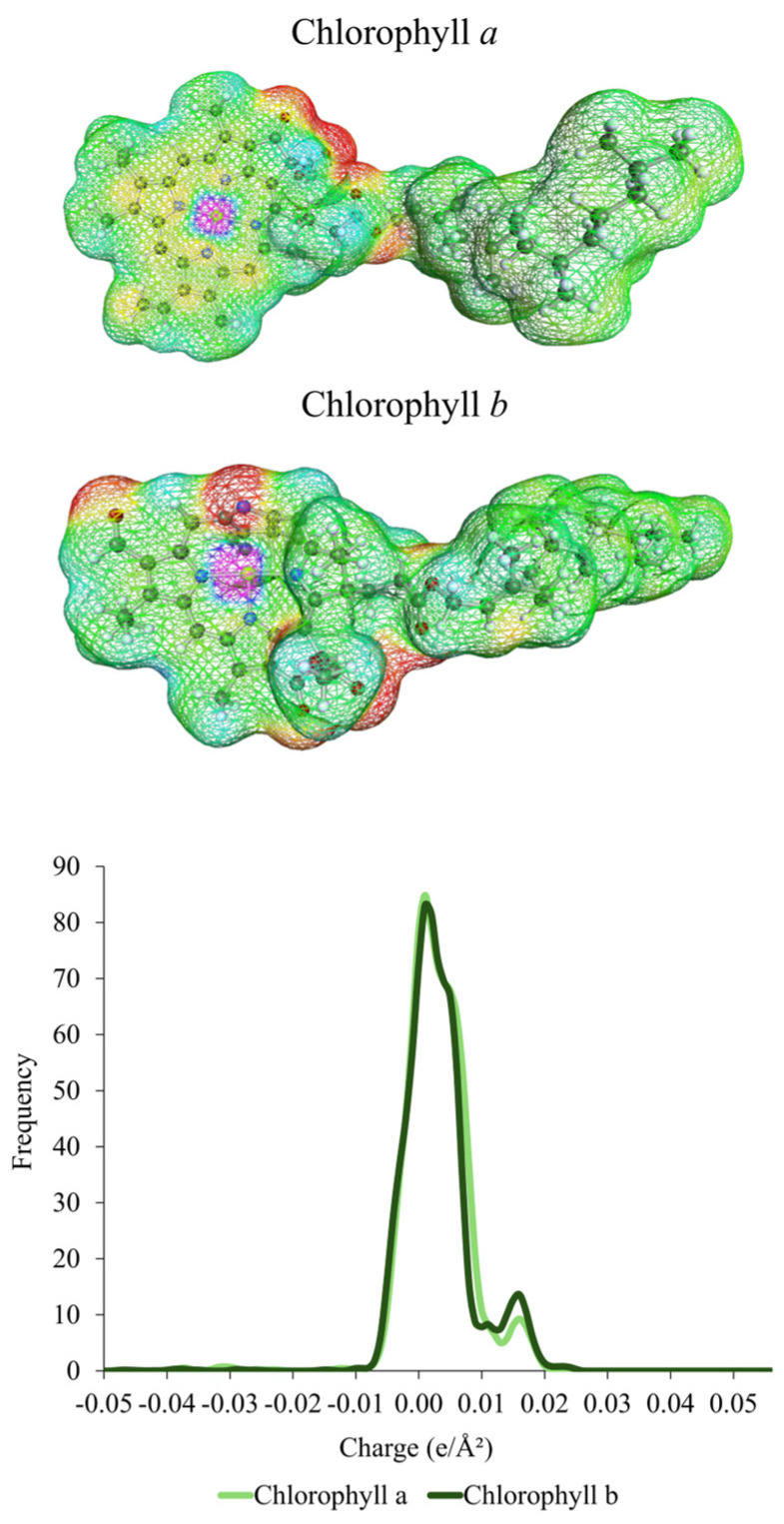
Charge density surface of chlorophylls *a*, *b*, and the σ-profiles generated.

To support the main hypothesis that the menthol/lauric acid (2:1) DES 2 interacts preferentially with chlorophyll *a* and *b* molecules, charge density surface and σ-profiles of the chlorophylls were generated and are presented in Figure 5.

**Figure 6:**
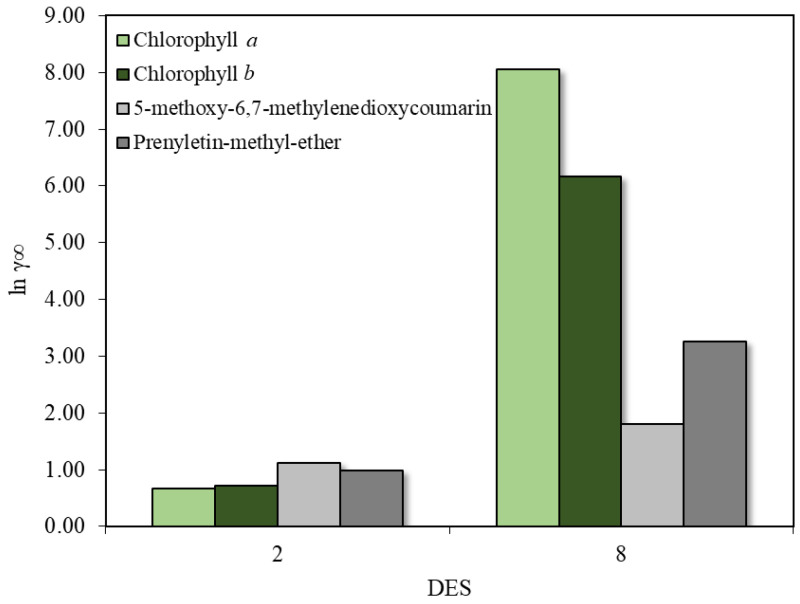
Absolute logarithmic activity coefficient at infinite dilution of chlorophyll *a*, chlorophyll *b*, 5-methoxy-6,7-methylenedioxycoumarin, and prenyletin-methyl-ether in DES 2 and DES 8 at 40 °C.

The chlorophyll *a* and *b* molecules contain long carbon chains in their structures, with large non-polar regions of neutrally charged zones. The intense peak in the non-polar region (0.00 e/Â^2^) of the σ-profiles was even more pronounced than for the coumarin molecules, highlighting the apolar characteristic of these pigments. Smaller positive-induction charge zones of oxygenated groups (red color) are also observed, while the only region of negative charge induction (blue color) due to the presence of magnesium is hindered by the carbon groups. As for the coumarin molecules, these results indicate a great affinity of the chlorophylls with lower polarity solvents, possibly even stronger. Therefore, a solubility screening of chlorophylls *a* and *b* in DES 2 and DES 8 at 313 K in COSMOThermX was performed, aiming to compare previous results of coumarin solubility and to theoretically demonstrate the preferential interaction of these pigments with DES 2 and DES 8, the best solvents in the screening part and in the extraction experiments, respectively, helping to explain discrepancies between experimental and predictive results. The estimated absolute logarithmic activity coefficient at infinite dilution of chlorophylls and coumarins is presented in Figure 6.

The results show a decrease in the logarithmic activity coefficient of the chlorophylls compared to the coumarins in DES 2, while they increase in DES 8. This agrees with the σ-profile analyses performed previously. For DES 8, the high logarithmic activity coefficient values estimated for chlorophylls indicate that during the extraction mechanism, the solvent will have an interaction preference and a certain selectivity for coumarin than chlorophyll molecules. On an individual level, DES 2 would be the solvent chosen to perform the extraction of coumarins and chlorophylls. In a scenario when coumarins and chlorophylls are competing as possible solutes, the ratio between logarithmic activity coefficients must be pair-wise. Table 3 helps visualize this scenario of solvent/solute preference interactions, where a relation between logarithmic activity coefficient of chlorophylls and coumarins in each DES was explored. The 5-methoxy-6,7-methylenedioxycoumarin and prenyletin-methyl-ether are represented as coumarin 1 and coumarin 2, respectively.

Values below 1.00 for all pair-wise combinations in DES 2 indicate a preferential interaction of the DES with chlorophylls, while values higher than 1.00 for all pair-wise combinations in DES 8 indicate preference of the DES for coumarin extraction. Even though DES 8 did not have the best result in the first coumarin solubility screening performed, the selectivity towards the target coumarins demonstrated here enhanced extraction efficiency and may be the reason why this DES showed the highest coumarin content in the UFLC analysis, while DES 2 preferentially interacted with pigments. This complex matrix and solute/solvent interaction needs to be evaluated at the beginning of the simulation with COSMO-RS for future studies to ensure a more accurate solvent screening. Another possibility to experimentally overcome this competition of target compounds and chlorophylls is to use a two-phase deep eutectic solvent system consisting of hydrophobic and hydrophilic DESs [39], resulting in two different types of extract.

### 2.6. Total Chlorophyll Content of the Extracts

The DES 2 and DES 8 extracts were also analyzed for total chlorophyll content. The results are presented in Table 4 as an average of three measurements.

Menthol-based DESs are expected to extract more fat-soluble molecules as chlorophylls than other DESs [24]. In fact, the highest chlorophyll amount was detected in DES 2, reaching 80.41 µg/g extract, almost twice the value found in DES 8, which, in turn, is in agreement with the solubility screening of chlorophylls previously performed. In addition to the hydrophobic nature of the DES, Ozel et al. [40] demonstrated that acidic conditions can enhance the amount of chlorophyll *a* extracted and, consequently, total chlorophyll content. DES 2 was reported to have an acidic pH [38], favoring the extraction mechanism.

## 3. Materials and Methods

### 3.1. DESs Screened

For this study, classic choline-chloride-based DESs and some previously reported DESs for the extraction of coumarins and other bioactive compounds [21,41] were selected as follows: choline chloride/glycerol (1:2), choline chloride/ethylene glycol (1:2), lactic acid/glycine/water (3:1:3), menthol/lauric acid (2:1), betaine/glycerol (1:2), betaine/glycerol (1:3), betaine/lactic acid (1:2), and betaine/ethylene glycol (1:3). All ratios are presented on a molar basis. These DESs can be formed and also differ in their physico-chemical properties such as density, viscosity, polarity, hydrophilic/hydrophobic character, and water content, as presented in our previous study [28], which is desirable in solvent screenings. In addition, all of these DESs have been reported before for phenolic extraction from *P. polystachyum* [28].

### 3.2. Solubility Screening in COSMOthermX

The solubility screening of 5-methoxy-6,7-methylenedioxycoumarin and prenyletin-methyl-ether in DES was performed in COSMOThermX (v. 22.0), a thermodynamic software that uses the *σ*-profiles of each component and the sum of the σ-profiles weighted with their mole fraction in the system to predict thermodynamic properties of compounds or mixtures. These coumarins, which present a potential therapeutic application, were selected because they have already been identified as the main compounds in the extracts of *P. polystachyum*, and no study applying computational prediction tools associated with these coumarins has been published to date. DESs were represented by the electroneutral mixture approach [42] with the HBA and HBD ratios previously defined, using the *σ*-profiles of each component available in the software database. To verify the adequacy of this approach, the density of all DESs at 313 K was predicted in COSMOThermX and compared with available experimental values from the literature.

The best DES to extract these coumarins from *P. polystachyum* was chosen according to the activity coefficient (${\gamma_{i}}^{\infty }$) at infinite dilution of the compounds in the solvents calculated in COSMOthermX (Equation (1)), which reveals the limit of the activity coefficient as the concentration of solute i approaches zero, where lower values mean more stability of the extract in the DES and, therefore, higher solubility [20].

(1) $$ln\left({\gamma_{i}}^{\infty }\right)=\frac{\left(\mu_{i}^{S,\infty }-\mu_{i}^{P}\right)}{RT}$$

The chemical potentials of pure compounds ($\mu_{i}^{P}$) and at infinite dilution ($\mu_{i}^{S,\infty }$) are determined from the *σ*-profiles, and the complete calculation methodology and equations used can be found elsewhere [43,44]. All calculations were performed with parameterization BP_TZVP_22.ctd and at 40 °C since it was reported as the optimal temperature for coumarin extraction [45].

### 3.3. Geometry Optimization Computation Details

For betaine, coumarin, and chlorophyll compounds, since they were not available in the COSMOthermX database, representation of the molecular structure was performed by Turbomole software (TmoleX v. 4.2). Ground state geometry optimization was performed using density functional theory (DFT) with the triple-zeta valence of polarization (def-TZVP) as the basis set and Beck–Perdew generalized gradient approximation (BP86) as the exchange-correlation functional. Turbomole software used single-point calculations to export the files with the ideal screening charges from each optimized molecular structure and created the σ-profiles. The files were then inserted into COSMOThermX software to predict thermodynamic properties and for the solubility estimation through the activity coefficient at infinite dilution calculation.

### 3.4. Experimental Deep Eutectic Solvent Extraction

#### 3.4.1. Plant Material

The *Pterocaulon polystachyum* plant material used in this study was previously reported by our group in previous studies [9,28] and was collected at Nova Santa Rita, Rio Grande do Sul, Brazil (29°54′01.7″ S 51°16′56.1″ W), during the February summer. Aerial parts (leaves and inflorescences) were separated from the stems and dried in an oven at 40 °C for 12 h, an essential step to avoid storage degradation due to water content in the plant matrix and to facilitate the grinding process. Subsequently, the plant material was milled and sieved, where the solid particles with a diameter of less than 0.63 mm were collected for the solid–liquid extraction experiments.

#### 3.4.2. DES Preparation

Four DESs selected based on coumarin solubility screening, namely menthol/lauric acid 2:1 (DES 2), choline chloride/glycerol 1:2 (DES 3), choline chloride/ethylene glycol 1:2 (DES 4) and betaine/ethylene glycol 1:3 (DES 8), were mixed using the heating and stirring method [46] for experimental deep eutectic solvent extraction. Their HBA and HBD compounds were weighed according to their determined molar ratios on an analytical balance and mixed under controlled temperature (323 K) until the formation of a clear and homogeneous solution without the generation of apparent crystals was achieved. All the prepared DESs were stored at room temperature for at least 24 h before extraction experiments. Table 5 provides detailed information about the components used in DES preparation.

### 3.5. Ultrasound-Assisted Extraction from P. polystachyum

Ultrasound-assisted extraction (UAE) has been reported to be associated with DESs as extraction solvents [47,48], enhancing the extraction efficiency [49] and enabling the process in shorter times than other extraction methods [50]. Therefore, to evaluate the selected DES ability to extract coumarins, UAE was performed using a combination of reported extraction parameters: 30 min, 313 K, and a solid/liquid (S/L) ratio of 1:20 [21,41]. A digital ultrasonic bath (Ultronique, Indaiatuba, Brazil, model Q 5.9/40 A) was used for UAE (40 kHz, 200 W, standard uncertainty *u*(*T*) = 3.00 K), a recommended methodology to be used with a large variety of samples under controlled temperature [51]. After the extraction, the samples were filtered to remove the solid residues, and the extracts were analyzed by ultrafast liquid chromatography and the spectrophotometric method.

### 3.6. Analysis of the Extracts

#### 3.6.1. Coumarins Analysis

The extracts were analyzed by ultrafast liquid chromatography (UFLC) according to the methodology reported by Medeiros-Neves et al. [34]. Briefly, 20 µL of each extract was mixed with 1 mL of acetonitrile and diluted in a solution of water and acetonitrile (1:1) until 10 mL. The samples were filtered using a suitable chromatograph filter (2.2 μm) before injection. A Shimadzu SPD-M20A HPLC (Kyoto, Japan) equipped with a diode array detector (DAD) and a Shim-pack XR ODS column (100 mm length, 2.0 mm internal diameter, and 2.2 μm particle size) and a C18 SecurityGuard™ ULTRA pre-column (Phenomenex, Torrance, CA, USA) were used. The analysis conditions were as follows: 5 μL of injection volume, 0.55 mL/min of flow rate for 8 min, temperature of 328 K, wavelength set to 327 nm, mobile phase composition as 0.1% formic acid (*v*/*v*), and acetonitrile and Multi-PDA Software (LC-solution Version 1.25 SP4) for monitoring and processing the output signal.

#### 3.6.2. Quantitative Analysis of Chlorophylls

Total chlorophyll content of the extracts was determined through UV-Vis spectrophotometric analysis, since a direct relation between absorbance and the amount of chlorophyll extracted was previously reported [36,40]. The methodology used was described by Tiago et al. [24], where 0.1 g of extract was diluted in 10 mL of ethanol 96% (*v*/*v*). Following dilution, samples were centrifuged for 15 min at 6.000 rpm, the supernatants were collected, and had their absorbance measured at 663 and 645 nm (Bel Engineering spectrophotometer, Monza, Italy, model UV-M51, standard uncertainty of wavelength less than 0.7 nm). The amount of chlorophyll *a*, chlorophyll *b*, and total chlorophyll content were calculated as follows:

(2) $$chlorophyll\;a=\left(12.25\times {Abs}_{663\;nm}\right)-(2.79\times {Abs}_{645\;nm})$$

(3) $$chlorophyll\;b=\left(21.50\times {Abs}_{645\;nm}\right)-(5.10\times {Abs}_{663\;nm})$$

(4) total chlorophyll=chlorophyll a+chlorophyll b

The constants of Equations (2)–(4) were reported for use with the DESs [24]. All the experiments were performed in triplicate, and the results were expressed as µg of total chlorophyll/g extract ± standard deviation.

## 4. Conclusions

This study helped to implement computational and predictive tools associated with natural products but highlights the need to assess the complexity of a plant matrix at the beginning of the simulation, especially to extrapolate the methodology to other plants and target compounds. COSMOthermX and Turbomole software could successfully represent the chemical structure of the molecules studied, and COSMO-RS proved to be an efficient model to predict the density of different DESs with less than 8% errors. DES 2 (Men/Lau 2:1) was indicated in coumarin solubility screening as the best solvent to obtain these compounds, but it was demonstrated that this solvent had a preference to interact with chlorophyll *a* and chlorophyll *b* during the extraction, therefore affecting the total amount of coumarins obtained. Predictive methodologies can help guide research and reduce experimental efforts, but they need to be evaluated in a larger context, especially for natural products, since the solvent is in contact and interacts with more compounds than the target one, which can influence the extraction mechanism predicted in the simulation and should be considered in the first steps of any study. New approaches considering the different solutes and solvent interactions, diffusion coefficients, and supramolecular structure of DESs can contribute to expanding the results presented.

## Figures and Tables

**Table 1:** Experimental and COSMO-RS predictive densities at 313 K for the DESs screened.

DES	No.	Density (g/mL)				
		Experimental	Ref.	COSMO-RS	Absolute Deviation *^a^*	Error (%) *^b^*
Lactic acid/Glycine/Water (3:1:3)	1	1.218	[21]	1.215	0.003	0.25
Menthol/Lauric Acid (2:1)	2	0.880	[21]	0.874	0.006	0.68
Choline Chloride/Glycerol (1:2)	3	1.169	[22]	1.146	0.033	2.80
Choline Chloride/Ethylene Glycol (1:2)	4	1.108	[23]	1.094	0.014	1.26
Betaine/Glycerol (1:2)	5	1.210	[24]	1.126	0.084	6.94
Betaine/Glycerol (1:3)	6	1.218	[24]	1.129	0.089	7.31
Betaine/Lactic Acid (1:2)	7	1.180	[25]	1.160	0.020	1.69
Betaine/Ethylene Glycol (1:3)	8	1.122	[24]	1.060	0.062	5.53

*^a^* Absolute deviation $=\left|\rho^{exp}-\rho^{pred}\right|$. *^b^* $Error\;\%=\left|\frac{d^{exp}-d^{pred}}{d^{exp}}\right|\times 100.$

**Table 2:** Peak A and B chromatographic area results of UFLC analysis of the DES extracts.

DES	No.	Peaks Area *^a^*	
		Peak A	Peak B
Menthol/Lauric Acid (2:1)	2	162.817	271.208
Choline Chloride/Glycerol (1:2)	3	55.031	155.797
Choline Chloride/Ethylene Glycol (1:2)	4	156.292	501.045
Betaine/Ethylene Glycol (1:3)	8	278.076	745.401

*^a^* Linearity between coumarin concentration and peak areas demonstrated elsewhere [34].

**Table 3:** Absolute logarithmic activity coefficient relation between chlorophylls and coumarins in DES 2 and DES 8.

$ln({\gamma_{i}}^{\infty })/ln({\gamma_{j}}^{\infty })$	DES 2	DES 8
Chlorophyll *a*/Coumarin 1 *^a^*	0.5869	4.4444
Chlorophyll *a*/Coumarin 2 *^b^*	0.6667	2.4693
Chlorophyll *b*/Coumarin 1 *^a^*	0.6314	3.4056
Chlorophyll *b*/Coumarin 2 *^b^*	0.7172	1.8922

*^a^* 5-methoxy-6,7-methylenedioxycoumarin. *^b^* Prenyletin-methyl-ether.

**Table 4:** Chlorophyll *a*, chlorophyll *b*, and total chlorophyll content of the DES extracts.

DES	No.	Chlorophyl *a* (µg/g Extract)	Chlorophyl *b* (µg/g Extract)	Total Chlorophyl (µg/g Extract)
Men/Lau (2:1)	2	32.06 ± 2.28	48.75 ± 6.15	80.41 ± 8.43
Bet/Et (1:3)	8	14.58 ± 1.34	30.55 ± 2.33	45.13 ± 3.67

The data are represented as the mean ± standard deviation of three measurements. Standard uncertainty *u* of wavelength less than 0.7 nm.

**Table 5:** CAS number, source, supplier purity, and molar mass of compounds used in DES formation.

Compound	CAS Number	Source	Supplier Purity *^a^*	Molar Mass (g/mol)
Choline Chloride *^b^*	67-48-1	Sigma-Aldrich	≥98.0%	139.6
Betaine	107-43-7	Sigma-Aldrich	≥98.0%	117.1
(DL)-Menthol	89-78-1	Sigma-Aldrich	≥95.0%	156.3
Ethylene Glycol	107-21-1	Sigma-Aldrich	≥99.5%	62.1
Glycerol	56-81-5	Sigma-Aldrich	≥99.5%	92.1
Lauric Acid	143-07-7	Sigma-Aldrich	≥98.0%	200.3

*^a^* Purity reported by the supplier. The reagents were used without further purification. *^b^* Stored in oven at 333 K owing to its hygroscopic nature. Sigma-Aldrich, Saint Louis, MO, USA.

## Data Availability

All the available data are reported in this work.

## Supplementary Materials

The following supporting information can be downloaded at: https://www.mdpi.com/article/10.3390/molecules30173468/s1, Figure S1: UFCL chromatogram profile of UAE DES 3 extract, highlighting peaks A (5-methoxy-6,7-methylenedioxycoumarin) and B (prenyletin-methyl-ether). Figure S2: UFCL chromatogram profile of UAE DES 4 extract, highlighting peaks A (5-methoxy-6,7-methylenedioxycoumarin) and B (prenyletin-methyl-ether). Figure S3: UFCL chromatogram profile of UAE DES 8 extract, highlighting peaks A (5-methoxy-6,7-methylenedioxycoumarin) and B (prenyletin-methyl-ether). Figure S4: UV absorption spectra of peaks A (5-methoxy-6,7-methylenedioxycoumarin) and B (prenyletin-methyl-ether) of the DES 3 extract. Figure S5: UV absorption spectra of peaks A (5-methoxy-6,7-methylenedioxycoumarin) and B (prenyletin-methyl-ether) of the DES 4 extract. Figure S6: UV absorption spectra of peaks A (5-methoxy-6,7-methylenedioxycoumarin) and B (prenyletin-methyl-ether) of the DES 8 extract.

## Abbreviations

The following abbreviations are used in this manuscript:

BP86	Beck–Perdew generalized gradient approximation
COSMO-RS	Conductor-like screening model for real solvents
DAD	Diode array detector
DES	Deep eutectic solvents
Def-TZVP	Triple-zeta valence of polarization
DFT	Density functional theory
HBA	Hydrogen-bond acceptor
HBD	Hydrogen-bond donors
UAE	Ultrasound-assisted extraction
UFLC	Ultrafast liquid chromatography
UV	Ultraviolet
