# Supplementary material for: COSMO-RS Solubility Screening and Coumarin Extraction from Pterocaulon polystachyum with Deep Eutectic Solvents
Source: Molecules. 2025 Aug 23;30(17):3468. doi: 10.3390/molecules30173468 (PMC12429901; doi:10.3390/molecules30173468)
Supplement: Supplementary file 1 [file molecules-30-03468-s001.zip › molecules-3796942-supplementary.pdf]

SUPPLEMENTARY MATERIAL

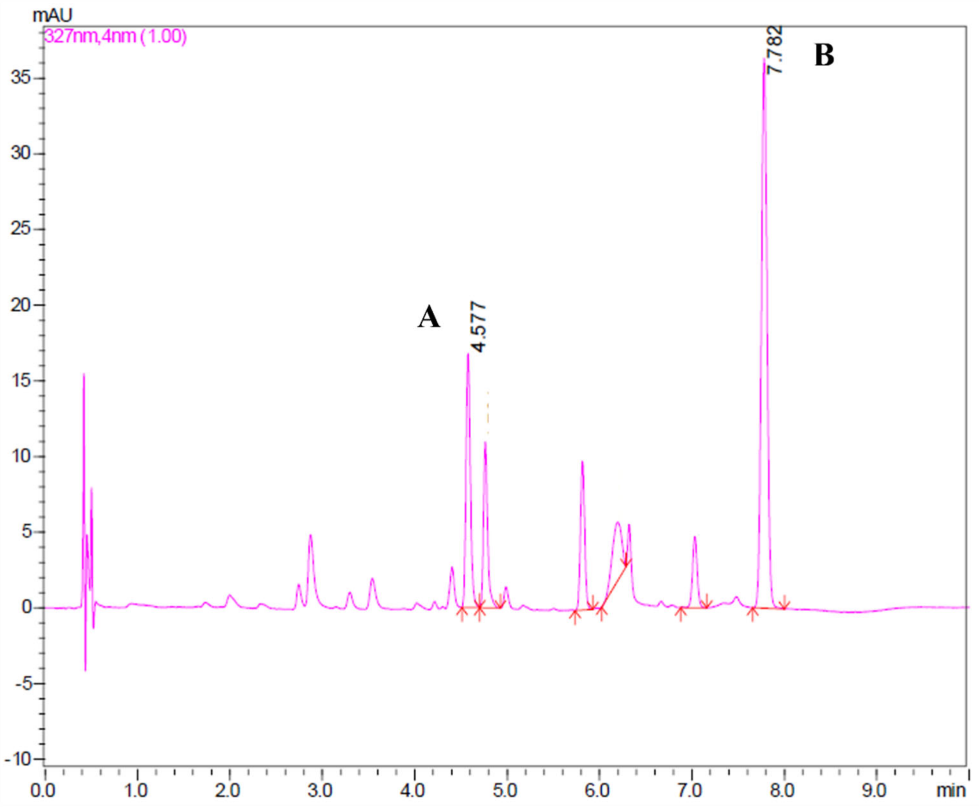

Fig S1 UFCL chromatogram profile of UAE DES 3 extract, highlighting peaks A (5-methoxy-6,7-methylenedioxycoumarin) and B (prenyletin-methyl-ether).

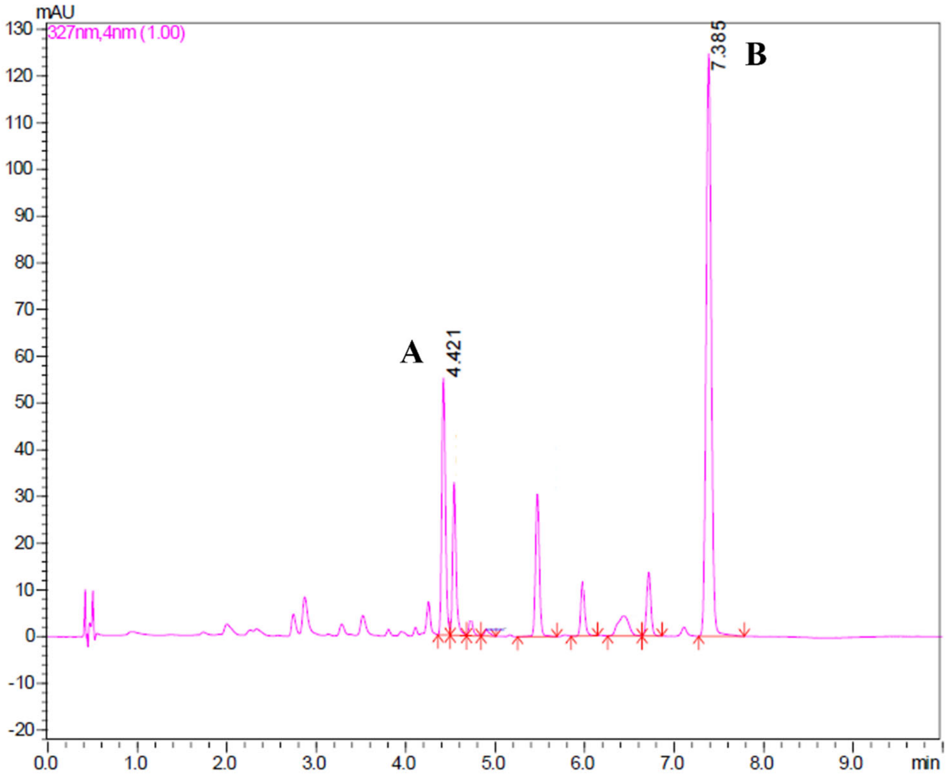

Fig S2 UFCL chromatogram profile of UAE DES 4 extract, highlighting peaks A (5-methoxy-6,7-methylenedioxycoumarin) and B (prenyletin-methyl-ether).

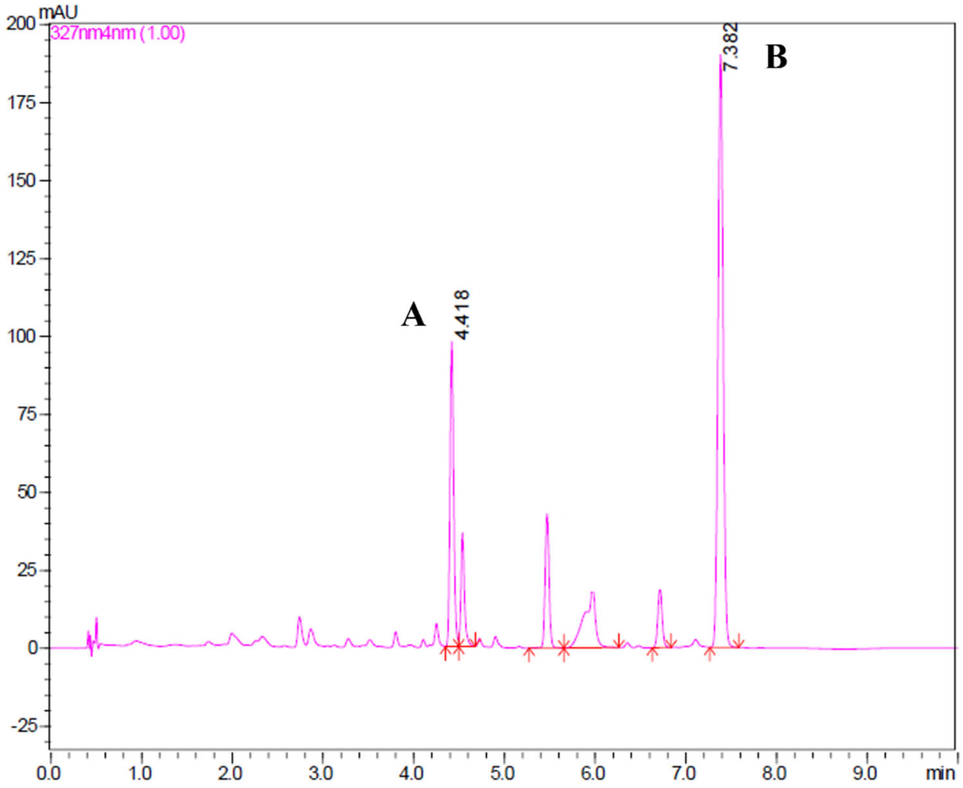

Fig S3 UFCL chromatogram profile of UAE DES 8 extract, highlighting peaks A (5-methoxy-6,7-methylenedioxycoumarin) and B (prenyletin-methyl-ether).

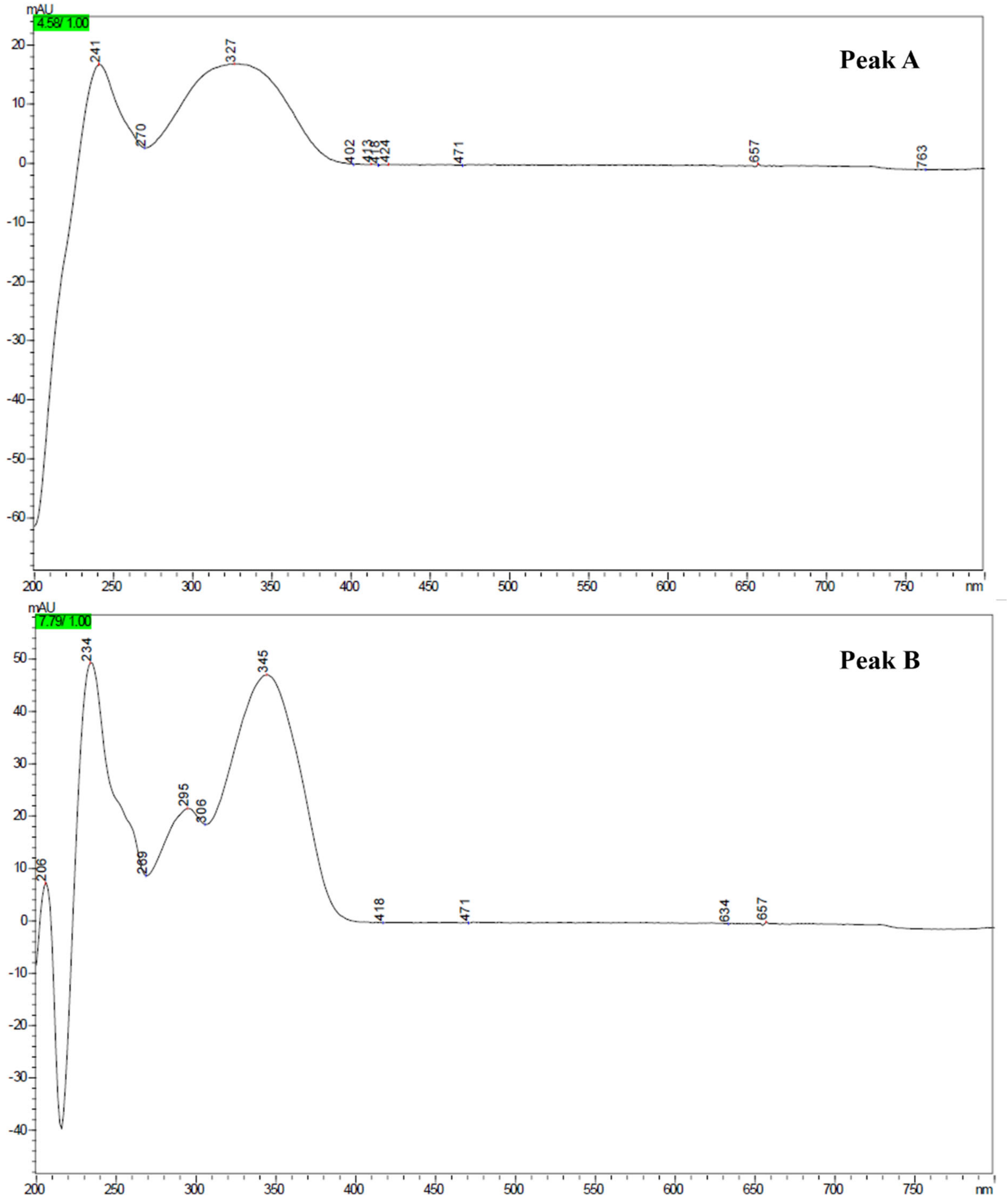

Fig S4 UV absorption spectra of peaks A (5-methoxy-6,7-methylenedioxycoumarin) and B (prenyletin-methyl-ether) of the DES 3 extract.

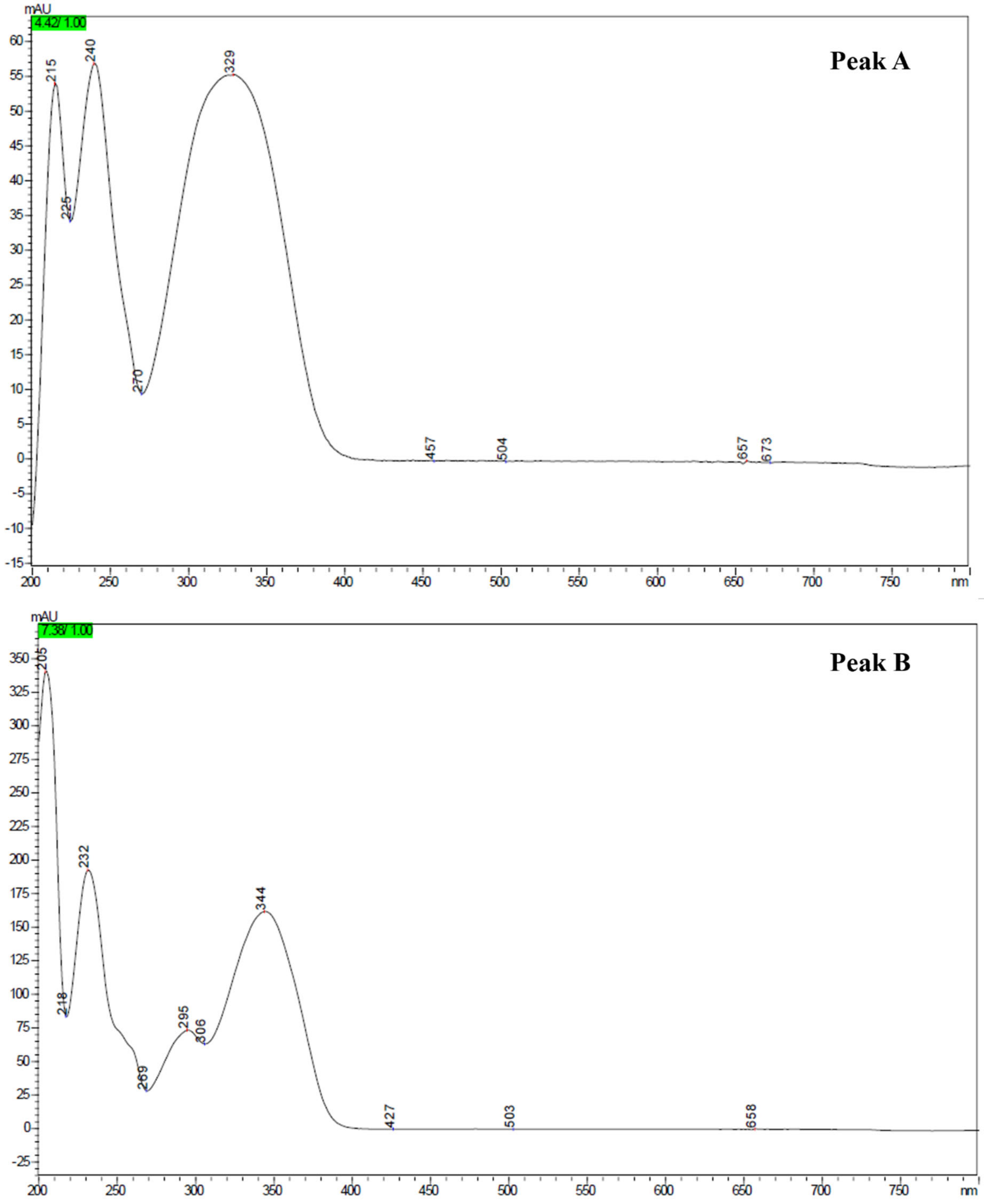

Fig S5 UV absorption spectra of peaks A (5-methoxy-6,7-methylenedioxycoumarin) and B (prenyletin-methyl-ether) of the DES 4 extract.

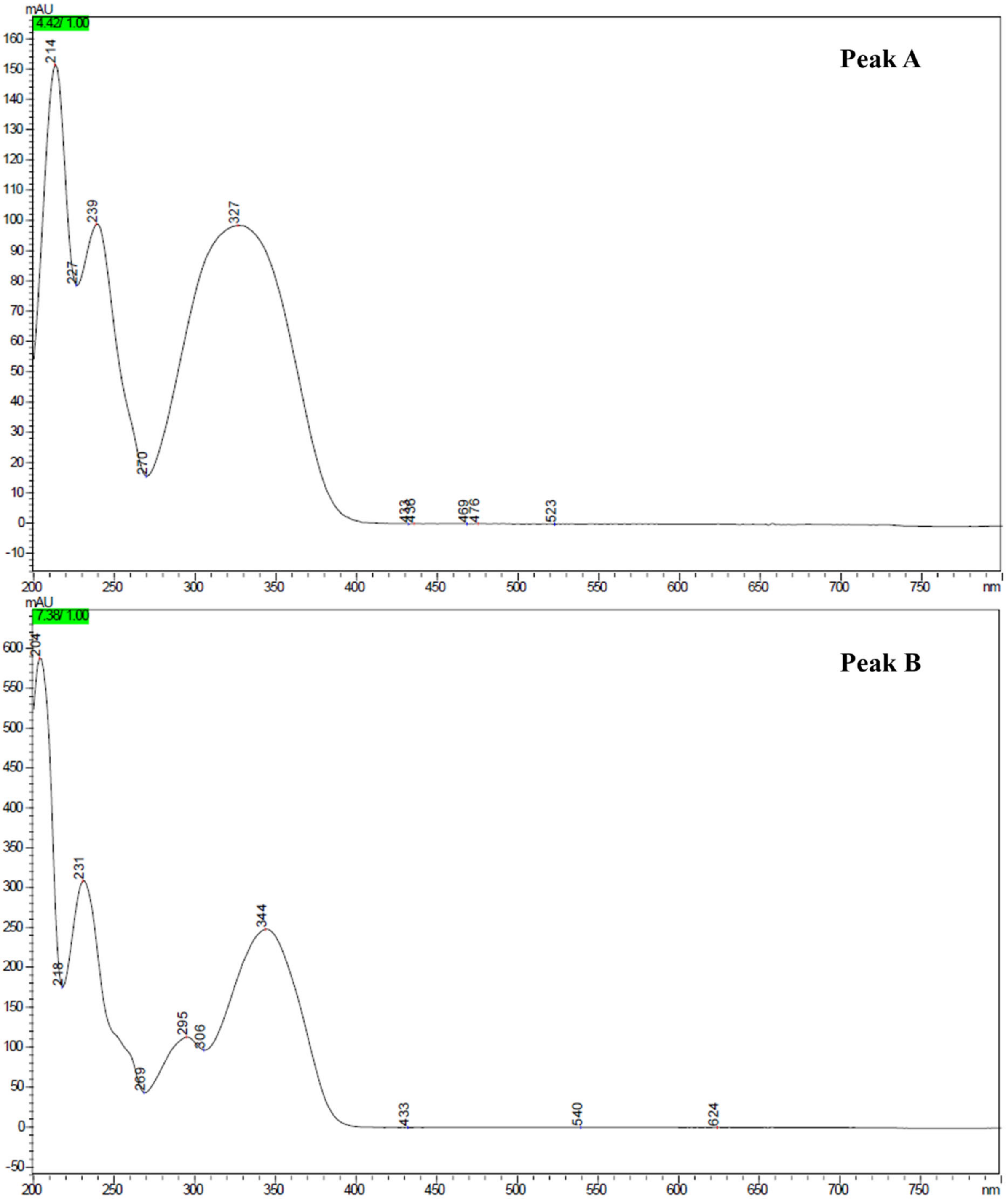

Fig S6 UV absorption spectra of peaks A (5-methoxy-6,7-methylenedioxycoumarin) and B (prenyletin-methyl-ether) of the DES 8 extract.
